# Meeting Highlights: Genome Informatics

**DOI:** 10.1002/cfg.300

**Published:** 2003-10

**Authors:** Jo Wixon, Jennifer Ashurst

**Affiliations:** 1 MRC UK HGMP-RC Hinxton, Cambridge CB10 1SB UK; 2 The Wellcome Trust Sanger Institute Wellcome Trust Genome Campus Hinxton, Cambridge CB10 1SA UK

## Abstract

We bring you the highlights of the second Joint Cold Spring Harbor Laboratory and Wellcome Trust ‘Genome Informatics’ Conference, organized by Ewan Birney, Suzanna Lewis and Lincoln Stein. There were sessions on *in silico* data discovery, comparative genomics, annotation pipelines, functional genomics and integrative biology. The conference included a keynote address by Sydney Brenner, who was awarded the 2002 Nobel Prize in Physiology or Medicine (jointly with John Sulston and H. Robert Horvitz) a month later.


**Comparative and Functional Genomics**
*Comp Funct Genom* 2003; **4**: 509–514.
Published online in Wiley InterScience (www.interscience.wiley.com). **DOI:** 10.1002/cfg.300

Feature

### Meeting Highlights: Genome Informatics

4–8 September 2002, Wellcome Trust Genome Campus, Hinxton, Cambridge, UK

Jo Wixon[1]* and Jennifer Ashurst[2]
[1]*MRC UK HGMP-RC, Hinxton, Cambridge CB10 1SB, UK*
[2]*The Wellcome Trust Sanger Institute, Wellcome Trust Genome Campus, Hinxton, Cambridge CB10 1SA, UK*

**Correspondence to:*
*Jo Wixon, MRC UK HGMP-RC, Hinxton, Cambridge CB10 1SB, UK.*
*E-mail: jwixon@hgmp.mrc.ac.uk*

### Abstract

**We bring you the highlights of the second Joint Cold Spring Harbor Laboratory and Wellcome Trust 'Genome Informatics' Conference, organized by Ewan Birney, Suzanna Lewis and Lincoln Stein. There were sessions on *in silico* data discovery, comparative genomics, annotation pipelines, functional genomics and integrative biology. The conference included a keynote address by Sydney Brenner, who was awarded the 2002 Nobel Prize in Physiology or Medicine (jointly with John Sulston and H. Robert Horvitz) a month later. Copyright © 2003 John Wiley & Sons, Ltd.**

### *In silico* data discovery

In the first of two sessions on this topic, **Naoya Hata (Cold Spring Harbor Laboratory, USA)** spoke about **motif searching for tissue specific promoters**. The first step in the process is to determine the foreground (positive) dataset and the background (negative) dataset and then search for over- or under-represented $n$-mers (where $n = 6–12$) in foreground sequences with respect to the background. Their tool can also be used to look for binding sites of dimers, by looking for two sequences (allowing for incomplete conservation) separated by $n$ nucleotides ($n = 0–12$).

They have accumulated data on 10 000 mouse promoters, from mouse Refseq and RIKEN cDNAs, and on 13 000 human promoters, from human Refseq and the database of transcription factor start sites (DBTSS website). Using data from a microarray study of the expression of ∼19 000 genes in 49 mouse tissues (Miki *et al.*, 2001), they identified 9000 data points with corresponding promoter sequences. They then tested their tool by trying to build a liver specific promoter database (LSPD).

Their foreground set was the promoter regions (an ∼1000 bp stretch upstream of genes) of those genes showing a log ratio of expression of >3 in liver compared to other tissues and their background set was genes which had a log ratio of ∼0 in liver. Their approach found 17 of 17 known promoters with a specificity of 17/28. None of the sites they identified was located downstream of a TSS and all showed an excess in the foreground sample compared to the background sample. They have also looked at muscle specific promoters and promoters specific for bone and kidney. So far, they see very little overlap in motifs between tissues, except for liver and kidney, which have several motifs in common. They hope to use this to build a discriminator function for tissue type.

**Klaus Hornischer (Biobase GMBH, Germany)** presented **a search for composite regulatory elements**. Applying a transcription factor binding motif search to an entire mammalian genome finds many hits (as expected, many are only 6-mers). One would expect to find a number of sites and elements in front of a gene, but enhancers also contain multiple sites, so the clusters of motifs that they observe are not always near to transcription start sites. His group have performed an analysis of clusters of sites observed on human chromosome 21. They found that the clusters were often at the start of genes and commonly showed high GC. They then classified the composite elements by function: inducible, constitutive or tissue-restricted. Using this approach, they see cross-coupling of functions

Copyright © 2003 John Wiley & Sons, Ltd.

(pathways) and can roughly predict the function or tissue role of a gene (they have several cases that match data on known proteins). This gives leads for expression experiments and functional analyses. A further benefit of this work is that the clusters can confirm gene models, or cause correction of models (typically elongation of models, or identification of missing 5′ UTRs).

Other talks in this session were given by Elena Rivas (Washington University, St Louis, USA) and Göran Sperber and Jonas Blomberg (Uppsala University, Sweden).

In the second session, **Uwe Ohler (MIT, USA)** presented work on **annotating the core promoter regions of *Drosophila* genes**. They used stringent criteria to cluster 5′ cap-trapped ESTs from the *Drosophila* Gene Collection, and then identified the transcription start sites (TSSs) of around 2000 genes. They then compared their dataset with *Drosophila* core promoter data from the Eukaryotic Promoter Database (EPD) and the core promoter database (CPD), finding good agreement for a number of criteria. Their search for motifs within these regions showed that a surprisingly low proportion of them contained binding sites for general transcription factors, such as TATA boxes. They also identified shared motifs that had not been described previously, which they then used to retrain their *ab initio* promoter prediction system (McPromoter), thereby enhancing its ability to recognize promoters (McPromoter prediction server).

**Abel Ureta-Vidal (EBI, UK)** described the **analysis and comparison of multiple genomes in EnsEMBL**. In the first step, they use 'exonerate' to compare DNA vs. DNA to locate synteny anchors. In the human vs. mouse comparison, 1 kb mouse fragments were located on the human genome by their best hit. Of these comparisons, ∼19 000 had informative high-scoring segment pairs (HSPs) and from these they selected very highly conserved regions. Just less than one-quarter of matches were in coding regions and around half were in intergenic regions, with the remainder in introns; the figures are roughly the same when looked at from the human or mouse perspective. For their protein level comparison, ∼20 000 human proteins and about the same number of mouse proteins were compared in an all vs. all search to find reciprocal best hits. While the majority of proteins found a good match, and so could be used as seeds (other genes are located with reference to these, based on genomic coordinates), significant numbers of paralogues, cuckoos (genes that have recently moved) and orphan proteins were also detected. The plan is to perform other comparisons of pairs of animal genomes (*C. elegans* vs. *C. briggsae* and *Drosophila* vs. the mosquito) and then to link them, but there are no plans to include plant genomes, as teams at other institutes already have this well in hand.

Other talks in this session were given by Damian Smedley (Imperial College, London, UK) and Heng Dai (Johnson & Johnson Pharmaceutical R&D, USA).

### Comparative genomics

**Orly Alter (Stanford University, USA)** described the application of generalized singular value decomposition to **comparing expression profiling datasets from two species**. They compared the Spellman *et al.* (1998) yeast cell cycle and the Whitfield *et al.* (2002) human cell cycle expression profiling datasets. Their results showed that the two datasets have the same gene patterns, but at different levels of significance. Human and yeast genelets with similar significance indicate common processes and they also found genelets exclusive to human or yeast, such as the yeast pheromone response genes. Plotting the data in circles by time showed the phases of cell division, with the expected patterns seen for known cell cycle-regulated genes. Even though the experiments were not synchronized at the same point, it was possible to see the conservation of phases; they were just out of step.

**Bin Liu (Baylor College of Medicine, USA)** discussed **a comparison of the human genome with draft sequences of mouse chromosome 11**. In the first phase of the project, a draft sequence for mouse chromosome 11 was constructed from the available data, which resulted in three large contigs and two gaps (which they believe to only be 2–3 BACs long). This was then compared to the human genome sequence. After clean-up of non-specific matches, they saw matches to almost every human chromosome. The largest block of homology is with human chromosome 17, and there are also significant blocks of homology with human chromosomes 7, 2, 5 and 22. About 7% of matches are in a mouse gene but outside of a human gene,

Copyright © 2003 John Wiley & Sons, Ltd. 

20% are in a human gene but outside of a mouse gene, 25% are non-genic in both species and the rest are gene–gene matches. He gave some detailed examples of the group's further work on specific regions. They have shown that the Smith–Magenis syndrome region is highly conserved in the mouse, most genes are in the same order, and they see some intergenic matches. In the p53–wnt3 inversion region they see variation of conservation across genes and some matches outside of genes, in particular some matches upstream of one gene, which could be its promoter. They have made mice with three different inversions of the syntenic region, which they plan to cross with ENU mutants.

**Aleksandar Milosavljevic (Baylor College of Medicine, USA)** presented the preliminary results of **comparative clone mapping and assembly of the Rhesus macaque and human genomes**. A pooled genomic indexing approach was first tested with rat BACs. An array of BACs is pooled and the pools are sequenced to ∼0.5× coverage. When a row pool and a column pool share the same human best hit, the intersection BAC is assigned to that location. A second set of pools (constructed from the same clones, but using a different design) is used to help eliminate false positives. For the Rhesus macaque they have 27 000 BACs, which gives ∼1.5× clone coverage. They have constructed pools of these and aim to sequence 144 reads/pool. Their comparative assembly approach uses the human assembly as a guide for the selection of BACs and for the assembly of the BAC sequences. A pilot study of this approach showed that it required over 20% less reads than using unassembled macaque sequences to achieve comparable assembly.

Other talks in this session were given by Seraphim Batzoglou (Stanford University, USA), Jo Dicks (John Innes Centre, UK), Irmtraud Meyer (Wellcome Trust Sanger Institute, UK) and Roman Tatusov (NCBI, USA).

### Annotation pipelines

**Robert Citek (Orion Genomics, USA)** presented **a system for managing a local copy of GenBank** on a PC (or even on a laptop, with a pared-down version of the database). To use it requires MySQL and selected Perl modules, BASH (or another UNIX-like shell) and ∼60 GB of space. The schema is just one table, with attributes of each entry. The sequence is held separately, in Perl-administered files. The set-up allows you to limit which parts of the database are used, e.g. viruses only. Taxonomy data is typically stored as an adjacency tree of parent to child, which makes it difficult to find descendants or ancestors. His system uses a nested set model, which allows fetching of all descendants of a parent, enabling searches for all vertebrate genes of a kind, for example. It is also possible to look at parsing errors (blank fields). He has found 68 000 cases (although many are molecule type, which is not a required field) and unexpected divisions (such as fungi in plants); the system has identified 115 000 of these. He also found other anomalies such as entries of less than 10 nucleotides in length, of which there seem to be several thousand (including 64 that are only one nucleotide long, some of which are N).

This talk prompted much discussion of how people use GenBank, which resulted in a show of hands that demonstrated that a significant proportion of the delegates prefer to hold a local copy. This was not seen in any way to reflect upon the service provided by NCBI, but rather to reflect on the reliability and speed of networks.

**James Galagan (Whitehead Institute, USA)** discussed the **annotation and analysis of the *Neurospora crassa* genome** using the CALHOUN system. This filamentous fungi is an important model, and has genome of ∼40 Mb spread across 7 chromosomes. They had 39.1 Mb in the current assembly, which had 833 contigs as ∼170 scaffolds. They combine three gene-calling tools, Fgenesh, Fgenesh+ and Genewise, which have different strengths, for the best results. They had 10 082 predicted protein coding genes at that time and were collaborating with over 30 members of the *N. crassa* and wider research community to analyse them. They have identified RIP which, during reproduction, detects duplicated sequences (including repeats, transposable elements, gene duplications and larger duplications) above a certain size with >80% sequence similarity, and mutates and thereby silences them. This is thought to be very important in the evolution of the *N. crassa* genome and may be widespread in fungi. Their multigene family analysis has shown that *N. crassa* has far fewer genes in families than would be expected from its genome size, when compared to other fungi, or to a broad range of species. Within those

Copyright © 2003 John Wiley & Sons, Ltd. 

families that do exist, there are very few highly similar gene pairs, i.e. it has almost no paralogues. This implies that it must have an alternative mechanism (to gene duplication) for gene evolution; he suggested perhaps gene sharing or lateral transfer, (although he pointed out that this is not widely documented in fungi). The work of the Fungal Genome Initiative should provide many more data that might help answer this question; they hope to have eight more fungal genomes in 2003 from the Whitehead Institute's efforts alone.

This session also included talks by Colin Weil (University of California, Berkeley, USA), Jeff Nie (Medical College of Wisconsin, USA), Feng Cao (Third Wave Technologies, USA), Carol Bult (Jackson Laboratory, USA), Michelle Clamp (EBI, UK) and John Quackenbush (TIGR, USA).

### Functional genomics

**Michael Eisen (Lawrence Berkeley National Laboratory, USA)** spoke about the **detection of transcription factor binding site motifs**. He explained that existing structurally aware motif detectors are all based on the EM algorithm and versions of the finite mixture model. They first developed CMEME, which uses motif family specific constraints on entropy curves to limit the shapes of motifs that it searches for. This approach performed better than MEME but was too slow to apply to a whole genome. A second approach, TF-EM, has positions constrained as highly, medium or weakly conserved and they specify a vector of constraints for each motif. This approach works faster than CMEME, but is still not ready to be applied to a whole genome. They have built contact maps from known protein–DNA complexes and incorporated these into the motif detection, by using TF-EM with a penalty for deviation from a specified profile. Using these methods, they have successfully found known *Drosophila* motifs, and have had good results with *Saccharomyces cerevisiae* binding sites. The software will eventually be available on one common platform, which will be open source.

**Michael Reich (Whitehead Institute, USA)** presented the next generation of **array analysis tools from the Whitehead Institute** Center for Genome Research (WICGR Cancer Genomics Software site). They have updated their popular data pre-processing and clustering tool, GeneCluster, which attracted 3000 downloads. The extra features of Version 2 include supervised classification, gene selection and permutation test methods. It has algorithms for building and testing supervised models using the weighted voting (WV) and $k$-nearest neighbours (KNN) algorithms, and has modules for batch SOM clustering and visualization. GeneCruiser is a new gene annotation tool that provides a quick, bidirectional link between Affymetrix probe IDs and gene information in public databases such as GenBank, UniGene and SwissProt. Users can also find out where Affymetrix probes are located in the human genome using the GoldenPath genome browser. The keyword search facility allows users to find out how many genes of a type (say, receptors) are represented by probes on each chip type. Their next generation pipelines will range from languages and object libraries for programmers who want to write their own pipelines to complete packages for users who prefer a 'black box' approach.

**Wyeth Wasserman (Karolinska Institute, Sweden)** presented work on **enhancing regulatory analysis using familial binding profiles of transcription factors** (TFs). Finding control signals by using genome-wide expression profiling followed by sensitive pattern discovery techniques to look for shared over-represented sequences in the control regions of co-regulated genes has been successfully applied to yeast, but has not been so successful for metazoan genomes. This new approach is based on the shared familial binding characteristics of TFs. The group developed a new algorithm for pairwise comparison of binding profiles and used this to align the profiles of well-known TF families to build family models for 11 major structural classes. They were able to use these models to predict the structural class of TFs acting via regulatory elements, and to enhance the detection of binding sites in metazoan promoter sequences. The approach is also less affected by the problems associated with analysing longer sequences.

There were also presentations by Peter Lee (McGill University, Canada), Y. Ramanathan (International Center for Public Health, USA), Xiaokang Pan (Cold Spring Harbor Laboratory, USA) and Jennifer Bryan (University of British Columbia, Canada).

Copyright © 2003 John Wiley & Sons, Ltd. 

### Integrative biology

The last session of the conference included a range of speakers involved with integrating informatics and annotation to obtain functional insights from genomic data. The session opened with **Kim Pruitt (NCBI, USA)**, who described projects concerned with **integrating sequence data with functional information extracted from PubMed**. The RefSeq project analyses transcripts through an automatic pipeline, followed by manual curation, to produce a high quality non-redundant resource for the genomic community. LocusLink GeneRIF incorporates functional data from PubMed abstracts into LocusLink. GeneRIFs can also be submitted by external users to aid the public annotation effort.

The **new Sanger Institute Gene Resources project** introduced by Jennifer Ashurst and Gareth Howell (Sanger Institute, UK) combines manual gene curation of individual chromosomes with experimental validation of the putative gene set, alongside extension of partial genes. Preliminary results from a pilot study of chromosome 20 annotation examined a total of 675 genes, 279 of which required experimental validation. Of these genes, 20% were confirmed with experimental evidence from cDNA pools and a further 20% of predictions had their structures changed when additional sequences were obtained.

**Simon Twigger (Medical College of Wisconsin, USA)** and **Fredrik Ståhl (Göteborg University, Sweden)** described the two official rat databases, both of which are involved in distributing rat gene nomenclature. **The Rat Genome database** (RGD) based in Wisconsin, uses the generic genome browser (**http://www.gmod.org**) to display quantitative trait loci (QTL), mouse and human comparative analysis, unigene data and microarray data, all mapped onto the genomic sequence. This enables the user to make connections from disease to QTL to gene. **RatMap**, which originates from Göteborg, concentrates on collecting and curating information about rat genes from literature sources and other databases. Over 1000 orthologues between mouse, rat and human genes have been curated and the database contains over 6000 new rat genes.

Producing structured vocabulary to describe biological annotations is a major goal for all model organism databases. **Judith Blake (MGI, Jackson Laboratory, USA)** described further **extensions to the Gene Ontology (GO) project**. Over 18 000 mouse genes have been curated using the primary literature and these can be queried using the standard ontology vocabulary. GO now includes the mouse anatomical dictionary and phenotype classification, which enables standardized annotation of gene expression and QTL analysis, and detailed description of experimental mouse mutants.

**Imre Vastrik (EBI, UK)** described **the Genome Knowledgebase** (GK), which utilizes information derived from the GO project. The GK project aims to capture all available information involved in cellular processes. These processes are broken down into two classes: Events and Physical Entities. Events consist of a series factors describing a process, which could include location, catalysts, inputs and outputs, etc. Alternatively, Physical Entities can be related to sequence accession numbers, GO identifiers for biological function, or biochemical activities. This should enable users to navigate easily through data involved in a particular process, e.g. DNA replication, and find all the genes, proteins and compounds involved in every step of that process.

There were also presentations by Junji Hashimoto (University of Tokyo, Japan) and David Block (Genomics Institute of the Novartis Research Foundation, USA).

### Keynote speaker

In his keynote speech, **Sydney Brenner** presented his view for 'the way ahead'. He feels that, while they have contributed much, bioinformatic approaches will not find everything that we want to know and that we cannot get all of the answers from the genome sequence. He proposed that research should now focus on cells, rather than the genome, with the aim of reconstructing pathways and understanding systems. He talked about his vision of **making a map of every cell** (commenting that histology studies indicate that there are ∼200 cell types in the human body) in terms of non-contingent entities, i.e. not those that are only expressed when cells are stimulated or stressed. He stated that the map would 'need to be accurate and complete; all databases should be like that', and commented that having standards would be

Copyright © 2003 John Wiley & Sons, Ltd.

important for the project. In answer to those who have responded that it is too complex, he argued that it is very rare for a protein to work alone in a cell, and suggested that the project is tackled as one component at a time (such as a ribosome, which would include ∼100 entities). The components would then become nodes in a giant graph that will be assembled. He has called his idea the 'instantiation program', where one instantiation is the expression of one form of a protein in a cell type, and the expression of a different form of the protein in the same, or a different, cell is another instantiation. The idea is then to take a cell and identify each instantiation of each protein in that cell. His group and others have shown that comparisons with *Fugu* genomic sequence can be used to find human and mouse promoters (which are what causes instantiation) as regions that have been conserved over time. Some *Fugu* promoters have been shown to work in the mouse, but he wants to also prove that they are necessary, and sufficient, for regulation. He is confident that all of his suggestions are possible, and expects the program to work by 2020, assuming that a large international project can be assembled.

### References

DBTSS website: **http://elmo.ims.u-tokyo.ac.jp/dbtss/**

McPromoter prediction server: **http://genes.mit.edu/McPromoter.html**

Miki R, Kadota K, Bono H, *et al.* 2001. Delineating developmental and metabolic pathways *in vivo* by expression profiling using the RIKEN set of 18 816 full-length enriched mouse cDNA arrays. *Proc Natl Acad Sci USA* **98**(5): 2199–2204.

Spellman PT, Sherlock G, Zhang MQ, *et al.* 1998. Comprehensive identification of cell cycle-regulated genes of the yeast *Saccharomyces cerevisiae* by microarray hybridization. *Mol Biol Cell* **9**(12): 3273–3297.

Whitfield ML, Sherlock G, Saldanha AJ, *et al.* 2002. Identification of genes periodically expressed in the human cell cycle and their expression in tumors. *Mol Biol Cell* **13**(6): 1977–2000.

WICGR Cancer Genomics Software site: **http://www.genome.wi.mit.edu/cancer/software/software.html**

---

The Meeting Highlights of *Comparative and Functional Genomics* aim to present a commentary on the topical issues in genomics studies presented at a conference. The Meeting Highlights represent a personal critical analysis of the current reports, which aims at providing implications for future genomics studies.

---

Copyright © 2003 John Wiley & Sons, Ltd.
